# Comparison of Cumulative Live Birth Rates Between Fresh and Vitrified Donor Oocytes

**DOI:** 10.7759/cureus.82589

**Published:** 2025-04-19

**Authors:** Kyparissia Kostoglou, Georgios Michos, Robert Najdecki, Ioannis Tsakiridis, Chartomatsidou Tatiana, Foteini Chouliara, Themistoklis I Dagklis, Apostolos M Mamopoulos, Evangelos Papanikolaou

**Affiliations:** 1 Private IVF Unit, Assisting Nature Centre of Reproduction and Genetics, Thessaloniki, GRC; 2 3rd Department of Obstetrics and Gynecology, Aristotle University of Thessaloniki, Thessaloniki, GRC; 3 Medical School, Aristotle University of Thessaloniki, Thessaloniki, GRC

**Keywords:** egg donation, fresh donor oocytes, frozen donor oocytes, icsi, live birth rates, oocyte vitrification

## Abstract

Background

Oocyte donation is a well-established treatment for age-related infertility, diminished ovarian reserve, and other reproductive challenges. The introduction of vitrification has expanded its feasibility by enabling oocyte cryobanking, reducing logistical constraints, and improving accessibility. However, debate persists regarding whether clinical outcomes differ between fresh and vitrified donor oocytes, particularly when embryos undergo double vitrification at both the oocyte and blastocyst stages. This study aims to compare cumulative live birth rates between fresh and cryopreserved donor oocytes in oocyte donation cycles.

Methodology

This retrospective cohort study analyzed 214 oocyte recipient cycles conducted at Assisting Nature Fertility Center from January 2018 to January 2023. Group A (n=112) received fresh donor oocytes, while Group B (n=102) received vitrified donor oocytes. Key outcomes included fertilization rate, blastulation rate, cumulative positive β-human chorionic gonadotropin rate, cumulative clinical pregnancy rate, and cumulative live birth rate. Statistical comparisons were performed using t-tests and chi-square tests, with significance set at p<0.05.

Results

Oocyte survival after thawing was 96%, with similar fertilization (87% vs. 84%) and blastulation rates (70% vs. 65%) between fresh and vitrified oocytes, respectively. Live birth rates after the first embryo transfer were comparable between Group A (59.8%) and Group B (58.8%; p>0.05). Cumulative live birth rates, including pregnancies from second and third embryo transfers, were 69.6% in Group A and 66.7% in Group B, with no statistically significant difference (p>0.05). Despite a slight trend toward lower initial pregnancy rates in the vitrified donor oocyte group, cumulative outcomes remained comparable between the two groups.

Conclusions

Both fresh and cryopreserved oocyte donation programs produce similar fertilization, blastulation, and cumulative live birth rates. The findings suggest that double vitrification (oocyte and embryo stages) does not negatively impact embryo viability or pregnancy success. Oocyte vitrification should be considered a routine option in oocyte donation programs if laboratories ensure their vitrification protocols yield outcomes comparable to fresh oocyte cycles.

## Introduction

In recent years, many women have delayed childbearing due to social and medical factors [1,2]. For those experiencing age-related infertility, oocyte donation offers a viable pathway [2,3]. This approach is also recommended for women with diminished ovarian reserve, those undergoing fertility preservation for cancer treatment, individuals with repeated in vitro fertilization (IVF) failure, and cases involving inherited genetic disorders [4,5].

Oocyte donation has become a well-established strategy in assisted reproductive technology (ART). Traditionally, donor-recipient synchronization was required, often necessitating international travel for treatment. While fresh oocyte donation remains a widely used IVF technique, it presents challenges such as donor cycle variability, unexpected complications affecting donor availability, and the logistical difficulty of synchronizing donors and recipients. The introduction of oocyte cryopreservation has significantly improved donation programs by enabling the establishment of oocyte banks. This advancement ensures immediate access to donor oocytes and reduces waiting times, as fresh donor eggs are often limited.

Despite the advantages of oocyte vitrification, debate persists regarding whether pregnancy outcomes differ between fresh and vitrified donor oocytes. Cryopreservation of gametes and embryos plays a crucial role in ART, with vitrification emerging as an alternative to slow freezing. The primary advantage of vitrification is minimizing ice crystal formation through high concentrations of cryoprotectants and rapid cooling rates. In this method, oocytes are plunged directly into liquid nitrogen, preventing the formation of intracellular and extracellular ice crystals [6-8]. Vitrification has demonstrated high post-warming survival rates and pregnancy and live birth rates comparable to those achieved with fresh oocytes [9-12].

While vitrified oocytes initially showed high survival rates, some studies report lower clinical pregnancy and live birth rates than fresh donor oocytes [13-16]. Conversely, other studies have found no significant differences in clinical pregnancy or implantation rates between fresh and vitrified donor oocytes [17]. However, these studies did not assess cumulative pregnancy rates, which account for the effects of double vitrification - once at the oocyte stage and again at the embryo stage for surplus embryos not used in the initial transfer. In these cases, embryos derived from cryopreserved donor oocytes were transferred during the first cycle, while the remaining embryos were vitrified for future use. As a result, further research is needed to determine whether pregnancy and live birth rates differ between embryos subjected to double vitrification and those cryopreserved only at the embryo stage.

This study aims to evaluate cumulative live birth rates in oocyte donation cycles by comparing fresh and vitrified donor oocytes. Group A consisted of patients who received fresh donor oocytes, while Group B included those who received cryopreserved donor oocytes. Additionally, fertilization and blastulation rates were compared between the two groups.

## Materials and methods

This cohort study evaluated the cumulative outcomes of oocyte donation cycles performed at Assisting Nature Fertility Center from January 2018 to January 2023. The study included 214 oocyte recipient cycles: 112 recipients received fresh donor oocytes (Group A), and 102 received frozen donor oocytes (Group B). In Group A, 956 mature metaphase II (MII) fresh donor oocytes were used. In Group B, 734 vitrified donor oocytes were thawed, of which 702 survived and were used for fertilization. Participants were selected from the oocyte donation program waiting list, and all were younger than 50 years. Each recipient provided informed consent before enrollment. The study design was approved by the Institutional Review Board, Ethical Committee of Assisting Nature, IVF Unit, Thessaloniki, Greece (Approval No. 03/2018).

The success rates of ART cycles were estimated cumulatively, incorporating results from the first and subsequent embryo transfers derived from a single fertilization cycle. The following outcomes were assessed for each group: fertilization rate, blastulation rate, cumulative positive β-human chorionic gonadotropin (β-hCG), cumulative clinical pregnancy rate, and cumulative live birth rate. The cumulative live birth rate included data from frozen embryo transfers (FETs) performed within the same donation cycle.

The oocyte donation program ensured donor and recipient anonymity, with the IVF unit maintaining full responsibility. Donor oocytes were allocated based on phenotypic and blood group compatibility, as mandated by Greek legislation. Donor eligibility criteria included age 20-32 years, body mass index <30 kg/m², anti-Müllerian hormone concentration ≥1.5 μg/L, regular menstrual cycles, adequate ovarian reserve, and no medical contraindications. All donors underwent psychological evaluation and transvaginal ultrasound to exclude polycystic ovaries, endometriosis, or other gynecological conditions.

Ovarian stimulation commenced on cycle day 2 with daily gonadotropins (175-300 IU). A fixed gonadotropin-releasing hormone antagonist protocol was initiated on stimulation day 6 with daily injections of ganirelix or cetrorelix (0.25 mg) [18-20]. Transvaginal ultrasound and blood testing were performed on day 6 and subsequently as needed. When ≥3 follicles reached ≥18 mm, final oocyte maturation was triggered with triptorelin (0.3 mg; triptorelin, Ipsen Biotech, Boulogne-Billancourt, France), and oocyte pickup was conducted 36 hours later [21,22]. Retrieved oocytes were either fertilized via intracytoplasmic sperm injection (ICSI) for fresh cycles or vitrified for later use. Fresh or frozen blastocyst transfer was performed after recipient endometrial preparation with estrogen and progesterone [8,23].

Retrieved oocytes were assessed for maturity, and only MII oocytes were included for fresh cycles or oocyte banking. Oocytes were incubated for two hours post-retrieval, then denuded chemically (via 30-second hyaluronidase exposure) and mechanically. Only MII oocytes with a visible polar body were used.

Oocytes for cryopreservation were vitrified, while the remaining oocytes were cultured for fresh cycles. All oocytes were fertilized via ICSI using either partner or donor sperm [20]. Blastocyst transfer occurred on days 5-6 of embryo development. Recipients in both groups received up to 10 MII oocytes per procedure.

Embryos were cultured in sequential medium (continuous single culture, Irvine Scientific, Santa Ana, CA) under controlled incubation conditions (6% CO₂, 5% O₂, 37 °C). On day 1, zygotes were assessed for the presence and morphology of two pronuclei (2PN) [24]. On day 3, embryo quality was evaluated based on cell number, blastomere symmetry, and fragmentation rate [19]. On day 5, blastocysts were graded using the Gardner and Schoolcraft scoring system [23]. Blastocyst quality was also evaluated before vitrification for FET cycles. Sperm preparation and ICSI followed the protocol described by Van Landuyt et al. [25].

Oocytes were cryopreserved on the day of retrieval, and blastocysts were cryopreserved on day 5 using the vitrification method described by Kuwayama et al. [24]. The vitrification kit from Kitazato (Tokyo, Japan) was used. Oocytes were equilibrated in a medium containing 7.5% (v/v) ethylene glycol and 7.5% (v/v) dimethyl sulfoxide (DMSO) for 15 minutes at room temperature. After a 1-minute wash in vitrification medium (15% ethylene glycol, 15% DMSO, and 0.5 M sucrose), oocytes were loaded onto a Cryotec (Cryotec Inc., Indianapolis, IN) straw and plunged into liquid nitrogen. Blastocysts were vitrified using the same protocol, with a 10-14 minute incubation in a vitrification medium [20].

A Kitazato warming kit was used for warming. Cryotec straws containing vitrified oocytes and blastocysts were removed from liquid nitrogen and placed in a thawing solution (20% HEPES ((4-(2-hydroxyethyl)-1-piperazineethanesulfonic acid)-buffered medium) at 37 °C for one minute. Oocytes and embryos were then transferred to a dilution solution (20% HEPES-buffered medium with 0.5 M sucrose) for three minutes, followed by two washes in a washing solution at room temperature for five minutes and one minute, respectively. Oocytes were then transferred to culture media. Survival was assessed via inverted microscopy, and viable oocytes were fertilized by ICSI two to three hours post-thawing [20].

Recipients undergoing FET followed an estrogen replacement protocol for endometrial preparation. Monitoring began on cycle day 2, with treatment initiated if estradiol was <80 pg/mL, progesterone was <1.5 ng/mL, and no ovarian cysts were detected. Estradiol (2 mg; Divina or Cyclacur) was taken at bedtime on day 2, increased to 4 mg on days 3-5, 6 mg on days 6-8, and 8 mg from day 9 onward. Estradiol was continued until pregnancy testing. After at least 10 days of estrogen use and confirmation of endometrial thickness ≥7 mm, micronized progesterone (200 mg; Utrogestan, Besins Healthcare, Le Concorde, Monaco) was administered vaginally three times daily, beginning six days before blastocyst transfer and continuing until pregnancy testing. If pregnancy was confirmed, progesterone was maintained until the 10th gestational week [17,18].

In Group B, fresh embryos derived from thawed oocytes were fertilized and transferred within the same cycle. The timing of oocyte thawing, fertilization, and embryo transfer was synchronized with the recipient’s cycle, following the same treatment protocol.

ART success rates were estimated as cumulative rates, defined as the probability of achieving a clinical pregnancy or live birth after a single stimulation cycle, incorporating all subsequent FETs. Given the increasing use of the “freeze-all” strategy, a modified success index was applied based on the Combined Fresh and Frozen Embryo Transfers per Individual (COMFFETI) method proposed by Papanikolaou et al. [19,20]. This binomial variable (yes/no) reflects whether a patient achieved pregnancy following ovarian stimulation.

A cumulative clinical pregnancy was recorded if pregnancy was achieved in either a fresh or frozen-thawed cycle derived from a single stimulation cycle. Similarly, a cumulative live birth was recorded if any FET cycle resulted in a live birth.

Statistical analyses were conducted using IBM SPSS Statistics for Windows, Version 19.0. (IBM Corp., Armonk, NY). Data were reported as mean ± standard deviation or percentages. Means were compared using a t-test, while proportions were analyzed using a chi-square test. A p-value <0.05 was considered statistically significant.

## Results

This study included 214 oocyte recipient cycles, divided into two groups based on the state of the donated oocytes: fresh or cryopreserved. A total of 112 (52.3%) patients received fresh donor oocytes, while 102 (47.7%) received cryopreserved oocytes. The statistical analysis examined fertilization rate, blastulation rate, cumulative positive β-hCG, cumulative clinical pregnancy rate, and cumulative live birth rate. Additional data were collected on the number of MII oocytes, 2PN embryos, and blastocysts. See Appendices.

The fertilization rate was calculated as the number of 2PN embryos divided by the total number of MII oocytes per case, while the blastulation rate was determined by the number of embryos reaching the blastocyst stage divided by the total number of 2PN embryos. Recipients of fresh donor oocytes typically received 6 to 10 MII oocytes, whereas those receiving cryopreserved donor oocytes received 6 to 8.

In the frozen donation cycles, 734 vitrified MII oocytes were thawed for 102 recipients, with a mean of 7.1 oocytes per recipient (range: 6-8). Of these, 702 oocytes survived (96%) and were fertilized via ICSI, resulting in a mean fertilization rate of 84%. By day 5, 392 embryos reached the blastocyst stage, yielding a blastulation rate of 65%. In the fresh donation cycles, 956 MII oocytes were inseminated via ICSI, resulting in a fertilization rate of 87%. A total of 579 embryos developed into blastocysts by day 5, with a blastulation rate of 70%. Statistical analysis revealed no significant differences between Group A (fresh oocytes) and Group B (frozen oocytes) regarding the number of MII oocytes, 2PN embryos, blastocysts, fertilization rate, or blastulation rate (p > 0.05). The details are presented in Table 1.

**Table 1:** Embryology data for fresh versus vitrified oocyte donation cycles ^a ^N/A, not applicable (fresh oocytes are not thawed). ^b ^n.s., not statistically significant (p > 0.05). The t-test was used. ^c ^n.s., not statistically significant (p > 0.05). The chi-square test was used. ^d ^statistically significant (p <0.05). The t-test was used. ^e ^Mean (SD) (95% CI) Abbreviations: 2PN, two-pronuclear; MII, metaphase II

Parameter	Fresh Oocytes (Group A, n=112)	Vitrified Oocytes (Group B, n=102)	P-Value
Total MII oocytes (per group)	956	734	N/A ^a^
Survived oocytes post-thaw (per group)	N/A ^a^	702	N/A ^a^
MII oocytes (per acceptor) ^e^	8.5 (0.56 ) (7.93 - 9.06)	7.1 (0.53) (6.56 - 7.63)	0.29 ^b^
2PN embryos (per acceptor) ^e^	7.4 (0.58) (6.81 – 7.95)	5.9 (0.37) (5.52 – 6.27)	0.22 ^c^
Blastocysts (per acceptor) ^e^	5.1 (0.35) (4.74 – 5.45)	3.8 (0.25) (3.54 -4.05)	0.09 ^d^
Fertilization rate (%)	87%	84%	0.54 ^b^
Blastulation rate (%)	70%	65%	0.45 ^b^

The positive β-hCG, clinical pregnancy, and live birth rates after the first embryo transfer were compared between the two groups. In Group A, 73.2% of patients had a positive β-hCG result, 69.6% achieved clinical pregnancy, and 59.8% had a live birth. In Group B, the rates were 64.7%, 62.7%, and 58.8%, respectively. The two groups showed similar outcomes, with no statistically significant differences in any of the three parameters (p > 0.05; Table 2).

**Table 2:** Positive β-hCG, clinical pregnancy, and live birth rates for first, second, and third ETs in fresh versus vitrified oocyte donation cycles Abbreviations: ET, embryo transfer, β-hCG, β-human chorionic gonadotropin. ^a^Indicates no successes among two recipients in the third ET

Outcome	First ET	Second ET	Third ET	First ET	Second ET	Third ET
	Group A (Fresh, n=112)	Group A (Subset of 44)	Group A (Subset of 13)	Group B (Vitrified, n=102)	Group B (Subset of 31)	Group B (Subset of 2)
Positive β-hCG (%)	73.2 (82/112)	34.1 (15/44)	30.7 (4/13)	64.7 (66/102)	51.6 (16/31)	0 (0/2)^a^
Clinical Pregnancy (%)	69.6 (78/112)	29.5 (13/44)	23.0 (3/13)	62.7 (64/102)	32.2 (10/31)	0 (0/2)^a^
Live Birth (%)	59.8 (67/112)	22.7 (10/44)	7.6 (1/13)	58.8 (60/102)	25.8 (8/31)	0 (0/2)^a^

Some recipients in both groups who did not achieve a live birth after the first embryo transfer proceeded to a second or third embryo transfer using cryopreserved blastocysts, if available. Recipients with pending cryopreserved embryos were not included in this analysis. In Group A, 44 of 112 recipients underwent a second embryo transfer, resulting in a 22.7% live birth rate (12/44). Of those, 13 recipients proceeded to a third embryo transfer, yielding an additional 7.6% live birth rate (13/112). In Group B, 31 of 102 recipients underwent a second embryo transfer, with a 25.8% live birth rate (13/31). Two recipients proceeded to a third embryo transfer, but neither achieved a live birth.

Cumulative success rates were estimated for both groups. In Group A, 81.2% of patients had a cumulative positive β-hCG result, compared to 72.5% in Group B. The cumulative clinical pregnancy rate was 78.6% in Group A and 68.6% in Group B. The cumulative live birth rate was 69.6% in Group A and 66.6% in Group B. Although the cumulative rates were higher in Group A, statistical analysis showed no significant differences between the two groups (p > 0.05; Table 3).

**Table 3:** Cumulative positive β-hCG, clinical pregnancy, and live birth rates in fresh versus vitrified oocyte donation cycles ^a ^n.s., not statistically significant (p>0.05). The chi-square test statistic was used. The numbers in [] represent 95% CI. Abbreviations: β-hCG, β-human chorionic gonadotropin

Outcome	Group A (Fresh, n=112)	Group B (Vitrified, n=102)	P-Value (chi-square)
Cumulative Positive β-hCG (%)	81.2% [74%, 88%] (91/112)	72.5% [63%, 81%] (74/102)	0.13 ^a ^^(2.28)^
Cumulative Clinical Pregnancy (%)	78.6% [70%, 85%] (88/112)	68.6% [59%, 77%] (70/102)	0.09 ^a ^^( 2.73)^
Cumulative Live Birth (%)	69.6% [61%, 77%] ( 78/112)	66.6% [57%, 75%] (68/102)	0.64 ^a^^ ( 0.21)^

## Discussion

Oocyte donation is a vital fertility option for women of advanced maternal age or those with ovarian failure. The introduction of oocyte vitrification has further increased its accessibility and feasibility. The present study demonstrated that both fresh and frozen oocyte donation methods yield comparable cumulative live birth rates, the primary outcome of IVF.

Cryopreserved donor oocytes simplify the logistical challenges of synchronizing donor and recipient cycles, facilitate the establishment of oocyte banks, and allow for long-distance shipment of frozen oocytes. Vitrification is an effective and safe cryopreservation method, but concerns remain regarding its potential impact on oocyte integrity. Improper handling during freezing may compromise the meiotic spindle or cause cellular and subcellular alterations [13]. The primary challenge in oocyte cryopreservation is preventing ice crystal formation, which can damage the plasma membrane.

Although several studies have reported similar fertilization, blastulation, and embryo morphology outcomes between vitrified and fresh donor oocytes [26], skepticism persists among some patients and fertility specialists. Our findings support these previous reports, showing a high survival rate (96%) for vitrified oocytes and strong blastocyst development. Fertilization rates (87% vs. 84%) and blastulation rates (70% vs. 65%) were similarly high in both the fresh and frozen groups, respectively. These results suggest that the vitrification protocol used in this study, implemented by experienced embryologists, enables successful frozen oocyte donation cycles through an efficient freezing-warming process [11,12].

This study was designed to assess whether cumulative live birth rates differ between two groups: one receiving frozen-thawed embryos derived from fresh donor oocytes and the other receiving fresh embryos derived from vitrified-thawed donor oocytes. This comparison sought to determine whether the double vitrification process in the frozen group (once at the oocyte stage and again at the blastocyst stage) negatively affected implantation potential. The first embryo transfer results showed comparable live birth rates between the two groups, with a trend toward higher live birth rates in the fresh donor oocyte group. Similar findings have been reported in other studies, indicating reduced live birth rates among vitrified oocyte recipients [27,28].

However, when cumulative pregnancies from second and third embryo transfers were included, no significant differences were observed between the two groups in cumulative live birth rates. These findings suggest that double cryopreservation (oocyte and embryo) does not adversely affect embryo survival, clinical pregnancy rates, ongoing pregnancy rates, or implantation potential.

One limitation of this study is that some recipients who delivered a child and had surplus cryopreserved embryos either discarded them, donated them, or chose not to undergo additional embryo transfers for personal reasons. However, this did not impact cumulative live birth rates, as these individuals were already considered to have achieved a live birth. Another potential limitation is that recipients of cryopreserved donor oocytes had fewer surplus embryos available for subsequent transfers. In fresh oocyte donation programs, the maturity rate of oocytes is unknown in advance, leading to the allocation of a slightly higher number of oocytes per donor. As a result, the fresh oocyte group had, on average, one more blastocyst available compared with the frozen group, which may explain the slightly higher cumulative clinical pregnancy rate in the fresh oocyte group, though this difference was not statistically significant.

## Conclusions

This study demonstrates that fresh and cryopreserved oocyte donation programs yield comparable outcomes in fertilization rate, blastulation rate, clinical pregnancy rate, and live birth rate, both after the first embryo transfer and cumulatively. Although a slight trend toward lower initial pregnancy rates was observed in the vitrified donor oocyte group, this difference was not statistically significant. Larger studies are needed to determine whether this trend has clinical relevance. The double vitrification process (at the oocyte and embryo stages) was highly effective under the specific conditions, protocols, and expertise of the embryologists in this study, resulting in successful cumulative live birth rates.

These findings reassure recipients that the origin of donor oocytes - fresh or vitrified - does not impact the overall likelihood of achieving pregnancy. Vitrification of donor oocytes should be considered a standard approach for oocyte donation and cryobanking, provided that each laboratory ensures its protocols and handling procedures yield outcomes comparable to those of fresh oocyte cycles.

## Appendices

**Table 4:** Embryology data for fresh versus vitrified oocyte donation cycles

Parameter	Fresh Oocytes (Group A, n=112)	Vitrified Oocytes (Group B, n=102)	P-Value
Total MII oocytes (per group)	956	734	N/A ^a^
Survived oocytes post-thaw (per group)	N/A^a^	702	N/A ^a^
MII oocytes (per acceptor) ^e^	8.5 (0.56) [7.93 - 9.06]	7.1 (0.53) [6.56 - 7.63]	0.29 ^b^
2PN embryos (per acceptor) ^e^	7.4 (0.58) [6.81 – 7.95]	5.9 (0.37) [5.52 – 6.27]	0.22 ^c^
Blastocysts (per acceptor) ^e^	5.1 (0.35) [4.74 – 5.45]	3.8 (0.25) [3.54 -4.05]	0.09 ^d^
Fertilization rate (%)	87%	84%	0.54 ^b^
Blastulation rate (%)	70%	65%	0.45 ^b^

**Table 5:** Positive β-hCG, clinical pregnancy, and live birth rates for first, second, and third ETs in fresh versus vitrified oocyte donation cycles β-hCG: β-human chorionic gonadotropin; ET: embryo transfer

Outcome	First ET	Second ET	Third ET	First ET	Second ET	Third ET
	Group A (Fresh, n=112)	Group A (Subset of 44)	Group A (Subset of 13)	Group B (Vitrified, n=102)	Group B (Subset of 31)	Group B (Subset of 2)
Positive β-hCG (%)	73.2 (82/112)	34.1 (15/44)	30.7 (4/13)	64.7 (66/102)	51.6 (16/31)	0 (0/2)^a^
Clinical Pregnancy (%)	69.6 (78/112)	29.5 (13/44)	23.0 (3/13)	62.7 (64/102)	32.2 (10/31)	0 (0/2)^a^
Live Birth (%)	59.8 (67/112)	22.7 (10/44)	7.6 (1/13)	58.8 (60/102)	25.8 (8/31)	0 (0/2)^a^

**Table 6:** Cumulative positive β-hCG, clinical pregnancy, and live birth rates in fresh versus vitrified oocyte donation cycles β-hCG: β-human chorionic gonadotropin

Outcome	Group A (Fresh, n=112)	Group B (Vitrified, n=102)	P-Value (chi-square)
Cumulative Positive β-hCG (%) 95% CI	81.2% [0.74, 0.88] (91/112)	72.5 % [0.63, 0.81] (74/102)	0.13 ^a^ ^(2.28)^
Cumulative Clinical Pregnancy (%) 95% CI	78.6% [0.7, 0.85] (88/112)	68.6 % [0.59, 0.77] (70/102)	0.09 ^a ^^(2.73)^
Cumulative Live Birth (%) 95% CI	69.6 % [0.61, 0.77] (78/112)	66.6 % [0.57, 0.75] (68/102)	0.64 ^a^ ^( 0.21)^
